# Low thyroxine serves as an upstream regulator of ecophysiological adaptations in Ansell’s mole-rats

**DOI:** 10.3389/fendo.2024.1329083

**Published:** 2024-03-19

**Authors:** Patricia Gerhardt, Sabine Begall, Caroline Frädrich, Kostja Renko, Alexandra Heinrich, Josef Köhrle, Yoshiyuki Henning

**Affiliations:** ^1^ Institute of Physiology, University Hospital Essen, University of Duisburg-Essen, Essen, Germany; ^2^ Department of General Zoology, Faculty of Biology, University of Duisburg-Essen, Essen, Germany; ^3^ Charité – Universitätsmedizin Berlin, corporate member of Freie Universität Berlin and Humboldt-Universität zu Berlin, Institut für Experimentelle Endokrinologie, Berlin, Germany; ^4^ German Federal Institute for Risk Assessment, German Centre for the Protection of Laboratory Animals (Bf3R), Berlin, Germany

**Keywords:** mole-rat, thermoregulation, metabolic rate, ecophysiology, thyroid hormone, subterranean habitat, African mole-rat

## Abstract

**Introduction:**

About 10% of all rodent species have evolved a subterranean way of life, although life in subterranean burrows is associated with harsh environmental conditions that would be lethal to most animals living above ground. Two key adaptations for survival in subterranean habitats are low resting metabolic rate (RMR) and core body temperature (T_b_). However, the upstream regulation of these traits was unknown thus far. Previously, we have reported exceptionally low concentrations of the thyroid hormone (TH) thyroxine (T4), and peculiarities in TH regulating mechanisms in two African mole-rat species, the naked mole-rat and the Ansell’s mole-rat.

**Methods:**

In the present study, we treated Ansell’s mole-rats with T4 for four weeks and analyzed treatment effects on the tissue and whole organism level with focus on metabolism and thermoregulation.

**Results:**

We found RMR to be upregulated by T4 treatment but not to the extent that was expected based on serum T4 concentrations. Our data point towards an extraordinary capability of Ansell’s mole-rats to effectively downregulate TH signaling at tissue level despite very high serum TH concentrations, which most likely explains the observed effects on RMR. On the other hand, body weight was decreased in T4-treated animals and T_b_ was upregulated by T4 treatment. Moreover, we found indications of the hypothalamus-pituitary-adrenal axis potentially influencing the treatment effects.

**Conclusion:**

Taken together, we provide the first experimental evidence that the low serum T4 concentrations of Ansell’s mole-rats serve as an upstream regulator of low RMR and Tb. Thus, our study contributes to a better understanding of the ecophysiological evolution of the subterranean lifestyle in African mole-rats.

## Introduction

Mammals inhabit nearly all terrestrial and aquatic habitats. Each habitat requires ecophysiological adaptations to cope with its ecological challenges. Rodents are the most species-rich order of mammals. Among the various habitats to which rodents have adapted, the subterranean habitat is presumably the least understood in terms of ecophysiological adaptations. Of the approximately 2,500 rodent species worldwide (1), about 250 species have evolved a subterranean lifestyle in which they inhabit self-constructed burrows that protect them from predators and fluctuating climatic conditions (2). However, life in the subterranean niche is also challenging due to harsh environmental conditions (3–5). As a physiological feature, many subterranean rodents have evolved a lower resting metabolic rate (RMR) as predicted by allometry (3, 6, 7). Three hypotheses serve to explain why a low RMR may have evolved as a convergent ecophysiological adaptation to cope with the environmental conditions found in underground burrows: Occasionally occurring hypoxic and hypercapnic conditions in tunnels and nest chambers [*respiratory stress hypothesis* (8)], high energetic costs for burrowing coupled with scarce food and water availability [*cost of burrowing hypothesis* (9)], and high risk of overheating in burrow systems, where heat dissipation through convective cooling and evaporation is less efficient [*thermal stress hypothesis* (10)]. The exact contribution of each of these aspects to the evolution of low RMR is still under debate, but most likely all three aspects synergistically exerted sufficient selective pressure to establish this key adaptation. Low core body temperature (T_b_) is another shared feature of many subterranean rodent species that is hypothesized to result from low RMR due to metabolic heat production known as obligatory thermogenesis. However, it is not clear whether the low T_b_ is simply a consequence of low RMR or the result of active downregulation in thermoregulatory tissues.

Thyroid hormones (THs), particularly the bioactive 3,3’,5-L-triiodothyronine (T3) and its prohormone thyroxine (T4), are major regulators of metabolic rate and energy turnover and therefore also of T_b_ via obligatory thermogenesis (11). THs increase RMR, facilitate the breakdown of energy stores, increase oxygen consumption and oxidative phosphorylation rates (11–13). On the other hand, THs can directly influence T_b_ by increasing thermogenesis: When the ambient temperature falls below the thermoneutral zone, obligatory thermogenesis is not sufficient to maintain T_b_. In this case, facultative thermogenesis increases heat production by inducing shivering thermogenesis in skeletal muscle and non-shivering thermogenesis in brown adipose tissue (BAT). To generate heat by non-shivering thermogenesis, uncoupling protein 1 (UCP1) in the inner mitochondrial membrane uncouples the re-entry of protons into the mitochondrial matrix from ATP production. To compensate for reduced ATP production, BAT attempts to restore the mitochondrial proton motive force by increasing lipolysis, which represents a thermogenic process resulting in heat dissipation (14). Although critically discussed, some evidence suggests that UCP3, expressed in BAT and skeletal muscle, is also involved in facultative thermogenesis (15–17). THs are able to induce the expression of *Ucp1* and *Ucp3* to increase heat production by non-shivering thermogenesis (11, 18).

Since THs are powerful homeostatic regulators, TH action is regulated at multiple levels. Secretion from the thyroid gland is mainly regulated by the hypothalamus-pituitary-thyroid-axis (HPT axis): thyrotropin-releasing hormone (TRH), secreted by the hypothalamus, stimulates the pituitary to secrete thyroid-stimulating hormone (TSH), which stimulates T4 and T3 secretion in the thyroid gland. The secretion of TSH and TRH underlies negative feedback control, which is regulated by circulating THs (19, 20). Less than 1% of the THs circulate in the blood as free hormones (fT4, fT3), because most are bound to transport proteins (21). At the cellular level, the uptake and efflux of THs in target tissues is promoted by several transmembrane transporters, such as monocarboxylate transporter 8 (MCT8) and organic anion transporter 1C1 (OATP1C1) (22). In target cells, three different iodothyronine deiodinases (DIOs) catalyze the deiodination of THs to either convert the prohormone T4 to the receptor-active T3 or convert T4 and T3 to inactive metabolites. While DIO2 exclusively catalyzes the conversion of T4 to T3, DIO3 exclusively inactivates T4 and T3 by converting either T4 to 3,3’,5’-T3 (reverse-T3, rT3) or T3 to 3,3’-T2. DIO1 is able to both activate and inactive THs by converting T4 to T3 or T4 to rT3 and rT3 to 3,3’-T2, although rT3 is the preferred substrate (23, 24). Only T3 can bind to the nuclear TH receptors TRα and TRβ (encoded by *Thra* and *Thrb*) and activate target gene expression (25).

Previously, we and others have demonstrated that subterranean African mole-rats, namely naked mole-rats (*Heterocephalus glaber*) and Ansell’s mole-rats (*Fukomys anselli*), have extremely low serum T4 concentrations, more similar to those of poikilothermic reptiles than mammals (26–28). Moreover, regulation of THs in these species showed many peculiarities (28). Typical of subterranean rodents inhabiting underground burrows, African mole-rats have a much lower RMR as predicted by allometry, and a low T_b_ like other subterranean rodents (6). It has been hypothesized that the unusual TH phenotype of African mole-rats is an important adaptation to the subterranean habitat to keep RMR and T_b_ low. To investigate the proximate and ultimate mechanisms of the mole-rat’s TH system, we treated Ansell’s mole-rats with T4 and conducted comprehensive analyses of the HPT axis, metabolic rate and thermoregulation on whole-organism and tissue level.

## Materials and methods

### Animal husbandry

All Ansell’s mole-rats were born, raised, and kept in the animal facility of the Department of General Zoology, University of Duisburg-Essen, Germany (see **Supplementary Table S1** for a detailed overview of the study animals). The animals were housed as family groups in glass terraria on wood shavings as bedding. Room temperature and humidity were kept constant at 26 ± 1°C and 40 ± 3%, respectively. The diet consisted of carrots and potatoes fed *ad libitum*, enriched with apples, cereals and lettuce once a week. Treated and untreated animals were weighed every day with an accuracy of 0.01 g. All animal experiments were conducted in accordance with the German Regulations for Laboratory Animal Science (GV-SOLAS) and were approved by the North Rhine-Westphalia State Environmental Agency (LANUV, no. 81-02.04.2019.A455).

### Thyroid hormone treatment and temperature logger implantation

15 non-reproductive Ansell’s mole-rats (5 females and 10 males) were randomly assigned to the treatment group receiving T4 (N=8) at a daily dose of 90 ng/g body weight (T2376, Sigma-Aldrich/Merck, Darmstadt, Germany) or the control group receiving vehicle solution (N=7) containing 15 mM NaOH, 50% propylene glycol, 5% BSA in PBS for 4 weeks (see **Supplementary Table S1** for a detailed overview of the study animals). The solutions were administered with subcutaneously implanted osmotic pumps (pump model 2001, ALZET®, DURECT Corporation, Cupertino, CA, USA) according to the manufacturer’s instructions. For implantation, we deeply anesthetized the animals with an intramuscular injection of 6 mg/kg ketamine (Ceva GmbH, Düsseldorf, Germany) and 2.5 mg/kg xylazine (Ceva GmbH) (29) and implanted the pumps subcutaneously through a small incision just below the scapulae. To measure T_b_ throughout the treatment period, we implanted temperature loggers (DST nano-T, Star-Oddi, Garðabær, Iceland) into the peritoneal cavity through a small incision at the abdominal wall. The data loggers were programmed to measure T_b_ at 20 min intervals throughout the whole treatment period. For three days, the animals received an analgesic (Carprofen 5 mg/kg s.c., Norbrook Laboratories, Newry, UK) twice a day and were isolated in a sterile terrarium for 24–48 h for recovery before being returned to their family group. At the end of the experiment, the animals were deeply anesthetized with an overdose of ketamine and xylazine and decapitated for tissue sampling.

### Infrared thermography

For non-invasive measurement of heat dissipation, infrared images of the dorsal and ventral sides were taken using a thermographic camera (Testo 875-1i, Testo AG, Lenzkirch, Germany) from 15 animals (vehicle-treated: n=7; T4-treated: n=8). The dorsal and ventral sides were defined as the surface excluding the head, neck, extremities, and tail. The shaved areas where the incisions were made for implantation of the osmotic pump and temperature logger were excluded from the analysis. Infrared images were generated and analyzed using IRsoft software (vers. 4.7, Testo).

### Measurement of resting metabolic rate

We determined resting metabolic rate of 15 animals (vehicle-treated: n=7; T4-treated: n=8) by measuring oxygen consumption at rest using an open-flow respirometry system according to our protocol described elsewhere (7). In brief, animals were food deprived before being placed individually in a custom-made stainless-steel chamber with an airtight acrylic lid. The chamber was immersed in a temperature-controlled water bath at a temperature of 29°C, which is within the thermoneutral zone of Ansell’s mole-rats (7). Ambient air was pushed through the chamber at a flow rate of 257 ± 3 ml/min regulated by a flow meter (model 35830, Analyt-MTC, Müllheim, Germany). Carbon dioxide and water were filtered from both the incurrent and excurrent air, and the oxygen content of the depleted air was measured with an oxygen sensor (Servomex Type 5200 Multi Purpose, Crowborough, UK) and recorded at 2 s intervals with DIAdem 8.0 (National Instruments, München, Germany). The experiment was terminated and repeated when a resting state was not observed after 2h. The lowest 150 consecutive readings (equal to 5 min of measurement) of oxygen consumption were used to calculate RMR by the following equation (30):


$$VO_{2}=\frac{F_{ri}\left(F_{i}O_{2}-F_{e}O_{2}\right)}{1-F_{e}O_{2}}$$

with: VO_2_= oxygen consumption (ml O_2_×min^-1^), F_ri_= incurrent flow rate (ml×h^-1^), F_i_O_2_=oxygen incurrent fractional concentration (%), F_e_O_2_= oxygen excurrent fractional concentration. All measurements were corrected for body weight, flow rate, and ambient air pressure.

### Quantification of serum thyroid hormone levels

Blood samples were collected by venipuncture from the forepaw under ketamine/xylazine anesthesia before and after treatment to measure TH serum levels. The samples were centrifuged at 10,000 *× g* at room temperature for serum collection. Serum aliquots were stored at −80°C until use. Serum levels of fT4, fT3, total T4 (TT4), total T3 (TT3), rT3, and cortisol were determined using commercial ELISA kits according to manufacturer’s instructions (DRG Diagnostics, Marburg, Germany: FT4 – EIA 2386; FT3 – EIA 2385; TT4 – EIA 4568; TT3 – EIA 4569; Cortisol – EIA 1887; BioVendor, Brno, CZ: rT3 – RCD029R). The intraassay coefficients of variation (CVs) measured in samples with low, medium, and high hormone concentrations were 1.2–7.2% for fT4, 3.3–11.0% for fT3, 3.6–5.2% for TT4, 3.6–6.6% for TT3, 3.2–8.1% for cortisol, and 2.4–8.1 for rT3. Interassay CVs measured in samples with low, medium, and high hormone concentrations were 7.9–10.8% for fT4, 3.1–4.9% for fT3, 4.9–8.1% for TT4, 5.2–6.7% for TT3, 6.5–7.7% for cortisol, and 7.0–11.5% for rT3. We focused mainly on paired analyses of pre- and post-treatment hormone concentrations. Since serum volume was limited, only individuals with a full hormone profile comprising pre- and post-treatment measurements could be included in the analysis (n=10; vehicle-treated: n=5; T4-treated animals: n=5). TT3 was measured only in post-treatment samples to calculate TT3/TT4 ratios.

### Histological examination of thyroid glands

Thyroid lobes of 7 animals (vehicle-treated: n=3; T4-treated: n=4) were dissected, fixed overnight in 4% PFA, dehydrated, and embedded in paraffin. Coronal sections of 5 µm were cut from entire lobes and the most central sections were HE-stained. The slides were scanned using a digital slide scanner (ScanScope, Leica, Wetzlar, Germany) at 40× magnification. For quantification of thyroid lobe size, lobes were sketched in Corel DRAW (vers. 21.1.0.643, Ottawa, Canada) to create a mask for lobe size. For quantification of follicle cell heights, we selected an area with ten representative follicles and averaged the cell heights of five randomly chosen follicle cells per follicle using ImageJ (vers. 1.52p). Follicle numbers per section were counted in ImageJ.

### Glycogen assay

We determined the amount of glycogen in liver (untreated: n=7; vehicle-treated: n=5; T4-treated: n=5) and skeletal muscle (untreated: n=7; vehicle-treated: n=5; T4-treated: n=5) using a commercial glycogen assay kit according to the manufacturer’s instructions (MAK016-1KT, Sigma-Aldrich, St. Louis, MO, USA). In brief, tissue samples were immediately snap-frozen in liquid nitrogen and stored at -80°C until use. For glycogen measurements, frozen samples were cut, weighed, transferred to 100 µL ice-cold ddH_2_O, and homogenized. Subsequently, samples were heated to 95°C for 5 min, and cellular debris was separated by centrifugation. Liver and muscle samples were diluted 1:80 and 1:20, respectively. Absorbance was measured at 570 nm.

### Quantitative real-time PCR

For gene expression analyses, tissues were dissected, immediately transferred to RNAlater (Invitrogen, Karlsruhe, Germany), incubated overnight at 4°C, and stored at -20°C until use. We extracted RNA with the ReliaPrepTM RNA Tissue Miniprep extraction kit (Z6111, Promega, Walldorf, Germany) according to the manufacturer’s instructions. RNA concentration and purity were determined using a BioTek Epoch microplate spectrophotometer (Winooski, VT, USA). Depending on the size of each organ, 100–1000 ng RNA was reverse transcribed using the M-MLV reverse transcriptase (M1701, Promega, Walldorf, Germany) and oligo-dT primers. We validated tissues, which are often not easy to dissect, in a PCR with tissue-specific markers (thyroid gland: *Nis*; brown adipose tissue: *Ucp1*; pituitary: *Tshb*). Gene expression levels were quantified by quantitative real-time PCR (qPCR) using Blue S’Green qPCR Mix (331416L, Biozym, Oldendorf, Germany) in an iQ5 cycler (BioRad, Hercules, CA, USA). Cycling conditions were 1 cycle of denaturation (95°C/2 min) followed by 40 cycles of amplification (95°C/5 sec; 60°C/30 sec) and 37 cycles of melting curve acquisition (60°C/10 sec). To standardize all measurements, we set crossing threshold at 200 RFU. We validated the target gene expression of three different housekeeping genes (*Hprt1, Rps13, Ppia*) known to be unaffected by THs (31–33). However, in the present study, all three reference genes showed significant differences in several tissues tested, so normalization to housekeeping genes was unreliable. Therefore, we normalized the gene expression levels to total cDNA levels of each specimen measured using the 1x dsDNA High Sensitivity Kit (Q33230, Invitrogen, Carlsbad, CA, USA), a fluorescence-based method for highly selective determination of double-stranded DNA in a concentration range from 10 pg/µL to 100 ng/µL. This assay is specifically designed for measurement using the Qubit Fluorometer (Invitrogen, Carlsbad, CA, USA), which is the gold standard for quantification of cDNA for next generation sequencing (34–36). The applicability of this method as a suitable alternative to housekeeping gene-based normalization was previously validated (28).

All qPCR reactions were normalized to a starting amount of 100 pg cDNA using Equation 1


$$c\left(cDNA\right)>100\;pg/\mu L\to Ct_{100pg}=Ct+log_{2}\left[c\left(cDNA\right)/0.1\right];$$

$$c\left(cDNA\right)<100\;pg/\mu L\to Ct_{100pg}=Ct-{\text{log}}_{2}\left[c\left(cDNA\right)/0.1\right]$$

To verify the normalization to cDNA concentration, we prepared three dilutions of two biological samples of known concentration, including the target dilution of 0.1 ng/µL, and measured gene expression levels of two housekeeping genes. Application of Equation 1 resulted in similar Ct-values as quantified in the target dilution (SD = ± 0.17).

To create more intuitive diagrams where higher values represent higher target gene expression (TGE), we subtracted the normalized Ct-value from the number of total PCR-cycles:


$$TGE_{100\;pg\;cDNA}=40-Ct_{100pg}$$

**Figure 1:**
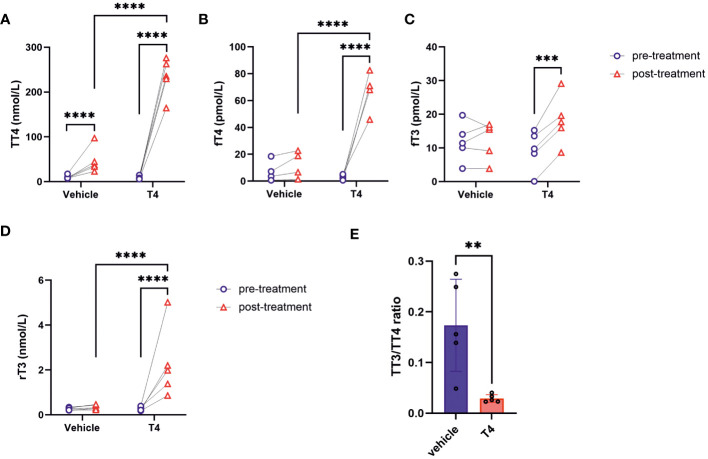
Thyroid hormone status of Ansell’s mole-rats. **(A)** TT4, **(B)** fT4, **(C)** fT3, and **(D)** rT3 concentrations of animals before and after treatment with vehicle or T4 solution measured by ELISA (Mixed-effects model for repeated measurements followed by Šídák correction for multiple comparisons, N=4–5). **(E)** Ratio of TT3 to TT4 reflecting the T4 to T3 conversion rate (Unpaired *t*-test, N=5). Data are presented as means ± SD. **p< 0.01, ***p< 0.001, and ****p< 0.0001. T4 – thyroxine, T3 – triiodothyronine, rT3 – reverse T3, TT4 – total T4, fT4 – free T4, TT3 – total T3, fT3 – free T3.

In addition to vehicle- and T4-treated animals (n=6 per treatment group; n=5 per treatment group for thyroid), we included gene expression data from untreated animals (n=7; n=6 for thyroid), to distinguish between T4 treatment effects and effects induced by pump implantation on serum TH concentrations, because we observed also significantly upregulated TT4 concentrations in vehicle-treated animals (see results; **Figure 1**). Gene expression of untreated Ansell’s mole-rats was compiled as part of a previous study (28) and are publicly available (37).

Primer pairs are listed in **Table 1**.

**Table 1:** Primer list.

target gene	amplicon length [bp]	5’ sequence	3’ sequence
*Dio1*	144	CAGGGCTATGGCTGAAGAGG	GATTCCCGGTCATCCCAGTC
*Dio2*	167	CCCGAAGAGGGAGACAGAGA	AGCACCCAGCAATAAGTCCC
*Dio3*	89	GAGCGCCTCTATGTCATCCA	CAGGTGCGAAGCTCTGAGAC
*Thra*	145	GCCAAGCAAAGTGGAGTGTG	CTGCTCGTCTTTGTCCAGGT
*Thrb*	208	CACCTGCTTGGCACTGTCTA	CAACCTGCGTGTCTTCCTCT
*Mct8*	146	GCTCCCTGTTCTACCACCAC	GCAGATTTGGGACCTGTGGA
*Oatp1c1*	252	GGAGCCGATGTGTGGAGAAA	GGAATGCCTCCAAGGGACAA
*Spot14*	107	CCTTACCCACCTCACCCAGA	CATCTGTGAAAAGGTCTCCCTTG
*Trh*	133	CACCTTGGCTGGAATACGTGA	TGGCGCTTCTTGGGTATTGG
*Tshb*	91	TGCCTGCTTTTTGGTCTTGC	AAGCACACTCTCGCCTATCG
*Ucp1*	93	TCAAGGGATTTGTGCCTTCCT	ACTTCGTCAGCTCTCGCTTC
*Ucp3*	180	GTGGGGCTATGGATGCCTAC	GGCAGGGGAAGTTGTCTGTTA
*Nis*	137	AGCCGCTACACTTTCTGGAC	TTGATGAGAACGGCCAGCTT
*Tpo*	181	CGATGCCTTCTTCAACCCCT	CAGGTTGAGTGATGCCAGGT
*Tshr*	218	ACGGACAACCCTTACATGACTT	AGTAGGGTCGGTCCACTGTA
*Serca1*	235	AGAGACCATCACCGCCTTTG	GGGGACTTTGTCTCCCACAG
*Serca2*	142	GAGCCCTTGCCACTCATCTT	CCAGTATTGCAGGTTCCAGGT
*Crh*	140	CACGTGCAGTGACAACACAA	TAGGGCGATTTCCGTCTGC
*Pomc*	195	GAGTTCAAGAGGGAGCTGACC	GTGGGAGTTCTTGAACAGCG
*Mc2r*	140	CTGCTTCAGAAAGTGGTAAGAACA	AGCTACTTGGGAGTGGTGATG
*Tg*	166	TCCAGTGCTTCAACTCGGAC	ACACTGGAGTTGACGAGCAG
*Hprt1*	83	CCAAAGATGGTCAAGGTCGC	TCAAACCCAACAAAGTCTGGC
*Rps13*	104	AGCCGGATTCACCGATTAGC	GACATTTATGCCACCAGGGC
*Ppia*	77	TCAACCCCTGCGTGTACTTC	GTCTGCAAACAGCTCGAAGG

### DIO1 & DEHAL activity

DIO1 activity measurement by determining iodide release from respective TH substrates using Sandell-Kolthoff-based readout has been described before (38). In brief, frozen tissues (n=6 per treatment group for liver and kidney; n=5 per treatment group for thyroid) were powdered in a microdismembrator. Tissue homogenates were prepared using homogenization buffer (pH 7.4, 250 mM D-(+) sucrose, 20 mM HEPES, 1 mM EDTA). Protein content was measured using Bradford reagent (BioRad). After adjusting protein concentrations, identical amounts of protein (60 µg for kidney and liver, 15 µg for thyroid tissue) were used to setup respective deiodination reactions. To subtract activities other than DIO1, background samples were spiked with 1 mM PTU. Subsequently, samples (100 mM KPO_4_ pH6.8, 40 mM DTT, 10 µM rT3) were incubated at 37°C for 2–3 h under constant shaking. Released iodide was separated from intact THs on a DOWEX50X2 column and subsequently quantified using a given iodide standard curve.

DEHAL activity was measured in a comparable setup to DIO1 for detecting enzyme-mediated iodide release, using mono-iodo-tyrosine (10 µM) as substrate. By using 15 µg protein for thyroid, and 80 µg protein from liver and kidney homogenate, with an incubation time of 2 (thyroid) or 4 (liver and kidney) hours, the DEHAL activity was determined as described before (28).

### Relative iodide content

10 µg of total thyroid protein (homogenate in 10 mM TrisHCl; n=5 per treatment group) was prepared in a total of 50 µl 0.6 M ammoniumpersulfate and digested in a thermocycler device at 90°C for 1 h. The resulting lysate was 10-fold diluted and 50 µl were transferred to the Sandell-Kolthoff-setup, as described above. OD at 405 nm was taken after 20 min of incubation as relative indicator of iodide content, as described before (39).

### Peroxidase activity

Peroxidase activity in the thyroids was measured by utilizing Amplex-UltraRed fluorescent dye (Invitrogen, Thermo Fisher Scientific) adapting the principle described for respective *in vitro* assays screenings (40) and applied before in mice (41). 5 µg protein per reaction from thyroid tissue homogenate (in 10 mM Tris-HCl; n=5 per treatment group) were transferred to a black microtiter plate (Brand, Wertheim, Germany). For each sample, two technical replicates were used for relative activity determination, with and without the addition of thiamazole (MMI) as thyroperoxidase (TPO) inhibitor (1 mM). Reaction was started by adding a master mix to reach assay conditions (100 mM KPO_4_ pH 7, 50 µM Amplex-UltraRed, 0.0035% H_2_O_2_) and subsequent incubation for 10 min at room temperature. Fluorescence was recorded (EX:535 nm, EM:590 nm) after shaking as readout for peroxidase activity within the samples in a plate reader (GENIOS, Tecan, Männedorf, Switzerland). The signal (RFU) from MMI-inhibited samples was subtracted from the respective uninhibited samples to calculate the relative MMI-sensitive peroxidase activity.

### Statistical analyses

Statistical analyses were performed using GraphPad Prism (vers. 9.3.1, San Diego, CA, USA). All data were tested for normal distribution using three normality tests (Anderson-Darling, D’Agostino-Pearson omnibus, and Shapiro Wilk) and validated using residual and QQ plots. Data were log-transformed if the datasets were not normally distributed. If normal distribution was not achieved by log-transformation, non-parametrical tests were applied. Paired and unpaired *t*-tests were applied to compare two groups, three groups were compared with a one-way ANOVA followed by Tukey’s multiple comparisons test or a Kruskal-Wallis test followed by Dunn’s multiple comparisons test when normal distribution was not achieved by log-transformation. Two-factor analyses were conducted with a mixed-effects model followed by Šídák correction for multiple comparisons. Additionally, core body temperature and heat dissipation data were analyzed with multiple linear regression models with treatment and sex as explanatory variables to control for sex effects, because we observed differences in treatment effects between females and males. These variables were used to calculate a full model with interactions. The Akaike information criterion (AIC) was used to estimate model fit. Model fit estimations and *p*-values were finally considered to estimate the biologically most relevant model. Significance level was set at α = 0.05. * < 0.05; ** < 0.01, *** < 0.001, and **** < 0.0001.

## Results

### Treatment effects on serum TH concentrations

We analyzed serum TH levels by means of ELISA. Baseline TH concentrations before hormone treatment with osmotic pumps were similar in all Ansell’s mole-rats (p>0.05). T4 treatment resulted in significant increase of serum TT4, fT4, and fT3 concentrations compared to pre-treatment concentrations (**Figures 1A-C**). Post-treatment fT3 concentrations did not differ between vehicle- and T4-treated animals, but as long as fT3 was increased compared to pre-treatment conditions, physiological effects can be expected. Only in T4-treated animals, fT3 concentrations were significantly higher after treatment compared to pre-treatment conditions. This was only observed in T4-treated animals. Unexpectedly, animals treated with vehicle-filled osmotic pumps also had significantly higher TT4 concentrations compared to pre-treatment concentrations, albeit approximately 5-fold lower than T4-treated animals (p<0.0001) (**Figure 1A**), suggesting that implantation of the osmotic pumps itself might have slightly triggered TH synthesis. Nevertheless, the TH concentrations of T4-treated animals showed much more pronounced treatment effects and the biologically-active free fractions of T3 and T4 were not influenced by vehicle treatment, emphasizing that the T4 treatment yielded the desired effects. Also, rT3 concentrations, a measure of T4 inactivation rates, were significantly increased only in T4-treated animals (**Figure 1D**). Additionally, the TT3 to TT4 ratio, a measure of TH activation rate, was significantly lower only in T4-treated animals (**Figure 1E**).

### Treatment effects on the HPT axis

**Figure 2:**
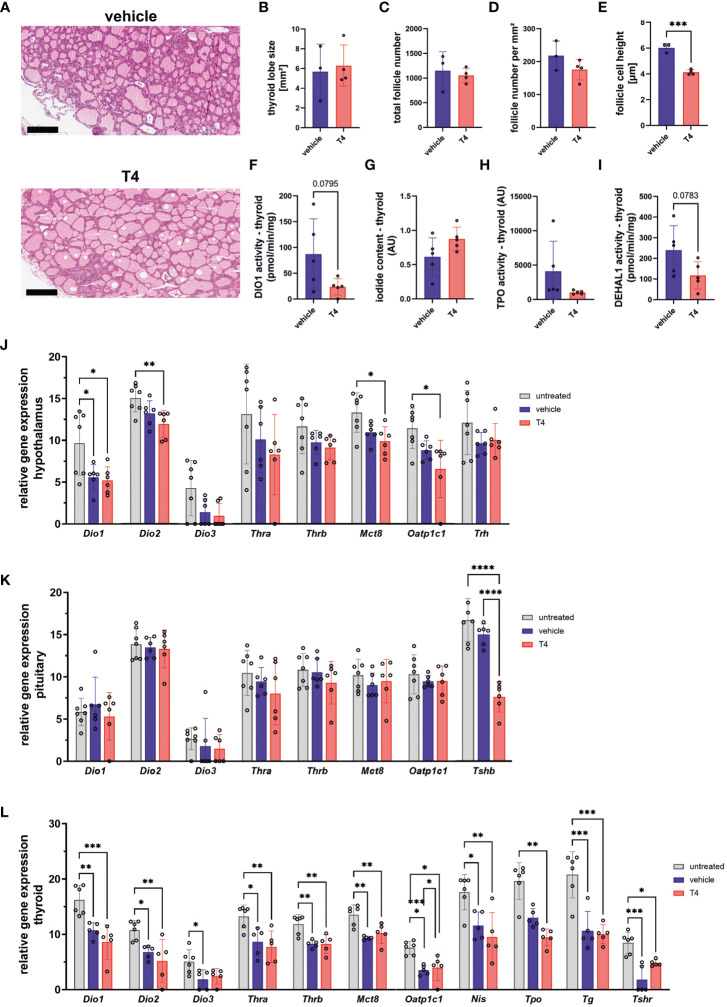
Treatment effects on the hypothalamus-pituitary-thyroid (HPT) axis. **(A)** Representative HE-stained PFA-fixed paraffin sections of thyroid glands from vehicle- and T4-treated animals. **(B)** Thyroid lobe size, **(C)** total number of follicles, **(D)** number of follicles per mm², and **(E)** height of follicle cells determined in HE-stained PFA-fixed paraffin sections from vehicle- and T4-treated animals (Unpaired *t*-test, N=3–4). **(F)** deiodinase 1 (DIO1) activity, **(G)** iodide content, **(H)** thyroperoxidase (TPO) activity, and **(I)** iodotyrosine dehalogenase 1 (DEHAL1) activity measured in thyroid glands from vehicle- and T4-treated animals (Unpaired *t*-test, N=5). Relative gene expression levels per 100 pg cDNA of **(J)** hypothalamus, **(K)** pituitary, and **(L)** thyroid gland from untreated, vehicle- and T4-treated animals (one-way ANOVA followed by Tukey’s multiple comparisons test or Kruskal-Wallis test followed by Dunn’s multiple comparisons test, N=6–7). Gene expression of untreated Ansell’s mole-rats was previously published (28) and is publicly available (37). Scale bar = 200 µm. Data are presented as means ± SD. *p ≤ 0.05, **p< 0.01, ***p< 0.001, and ****p< 0.0001.

Next, we analyzed the morphology of the thyroid gland in vehicle- and T4-treated animals (**Figure 2A**). We found no differences in thyroid size, total follicle number, or follicle number per mm² (**Figures 2B-D**), but the height of follicular cells was significantly decreased in T4-treated animals compared with vehicle-treated animals (**Figure 2E**).

We further measured thyroid DIO1 activity, iodide content, peroxidase activity potentially representing TPO (41), and iodotyrosine dehalogenase 1 (DEHAL1) activity to characterize thyroid function (**Figures 2F-I**). DEHAL1 is responsible for recycling iodide from iodinated tyrosine residues and TPO is responsible for oxidation of iodide to iodine, an essential step in TH synthesis. While DIO1 activity, iodide content, and TPO activity did not differ between groups (**Figures 2F-H**), DEHAL1 activity showed a trend towards lower activity in T4-treated animals (*p*=0.078; **Figure 2I**).

Treatment effects on gene expression level in the hypothalamus, pituitary, and thyroid which include several components involved in TH regulation, were further analyzed. In the hypothalamus, the expression of *Dio2, Mct8*, and *Oatp1c1* was downregulated exclusively in T4-treated animals compared with untreated animals (**Figure 2J**). *Tshb* expression in the pituitary, coding for the β-subunit of TSH, was also downregulated in T4-treated animals compared with vehicle-treated and untreated animals, indicating negative feedback regulation of the HPT axis (**Figure 2K**). In the thyroid, most genes tested were downregulated in vehicle- and T4-treated animals compared with untreated animals, except for *Tpo*, which was significantly lower in T4-treated animals compared to untreated animals (**Figure 2L**).

### Treatment effects on metabolic parameters

**Figure 3:**
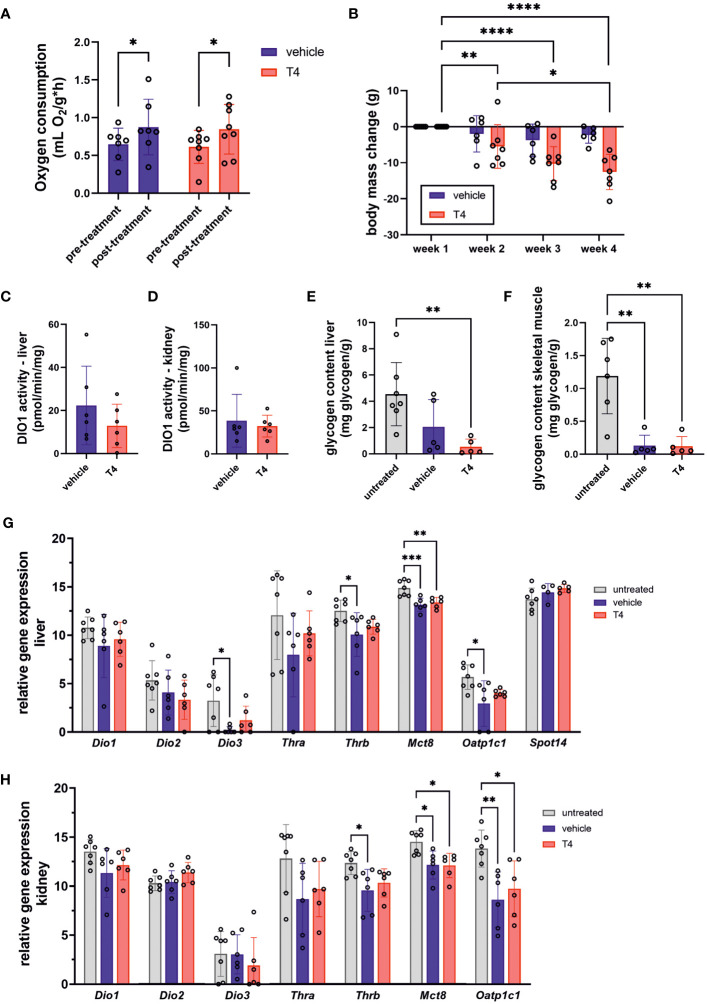
Treatment effects on parameters associated with regulation of metabolic rate and body weight. **(A)** mass-specific oxygen consumption of vehicle- and T4-treated animals before and after treatment calculated by normalizing oxygen consumption to body mass (Mixed-effects model followed by Šídák correction for multiple comparisons, N=7). **(B)** Body mass change during four weeks of treatment with vehicle or T4 solution calculated by subtracting individual baseline body weight determined on day 0 from weekly body mass (Mixed-effects model for repeated measurements followed by Šídák correction for multiple comparisons, N=7–8). **(C)** Deiodinase 1 (DIO1) activity in liver and **(D)** kidney of vehicle- and T4-treated animals representing two metabolically active organs (Unpaired *t*-test, N=6). Glycogen content of **(E)** liver and **(F)** skeletal muscle per g tissue of untreated, vehicle- and T4-treated animals (one-way ANOVA followed by Tukey’s multiple comparisons test, N=5–7). Relative gene expression levels per 100 pg cDNA in **(G)** liver and **(H)** kidney of untreated, vehicle- and T4-treated animals (one-way ANOVA followed by Tukey’s multiple comparisons test or Kruskal-Wallis test followed by Dunn’s multiple comparisons test, N=6–7, except for *Spot14*: N=4–7). Gene expression of untreated Ansell’s mole-rats was previously published (28) and is publicly available (37). Data are presented as means ± SD. *p ≤ 0.05, **p< 0.01, ***p< 0.001, and ****p< 0.0001.

THs are positive regulators of metabolic rate and energy turnover in virtually all animals. Thus, we hypothesized that T4 treatment would result in higher RMR in Ansell’s mole-rats and determined mass-specific RMR (msRMR) by indirect calorimetry in vehicle- and T4-treated animals before and after treatment. Unexpectedly, oxygen consumption was significantly increased by approximately 1.4-fold after treatment in both groups. Consequently, we found no significant difference in post-treatment oxygen consumption (**Figure 3A**). Although msRMR was unaffected, body weight was significantly reduced in T4-treated animals (**Figure 3B**), suggesting that overall energy turnover was increased compared to vehicle-treated animals. We further analyzed DIO1 activity in liver and kidney, two highly metabolically active organs, as DIO1 can convert T4 into the inactive rT3 and is also a T3 target gene. The activities measured in vehicle- and T4-treated animals did not significantly differ from each other (**Figures 3C, D**). In line with these findings, glycogen levels in liver and skeletal muscle did not differ significantly between vehicle- and T4-treated animals, albeit being lower compared to untreated animals (**Figures 3E, F**). Treatment effects on hepatic and renal gene expression were only observed for *Mct8* as well as *Mct8* and *Oatp1c1*, respectively, while the T3 target genes *Dio1* and *Spot14* were not affected by T4 treatment (**Figures 3G, H**).

### Treatment effects on thermoregulation

**Figure 4:**
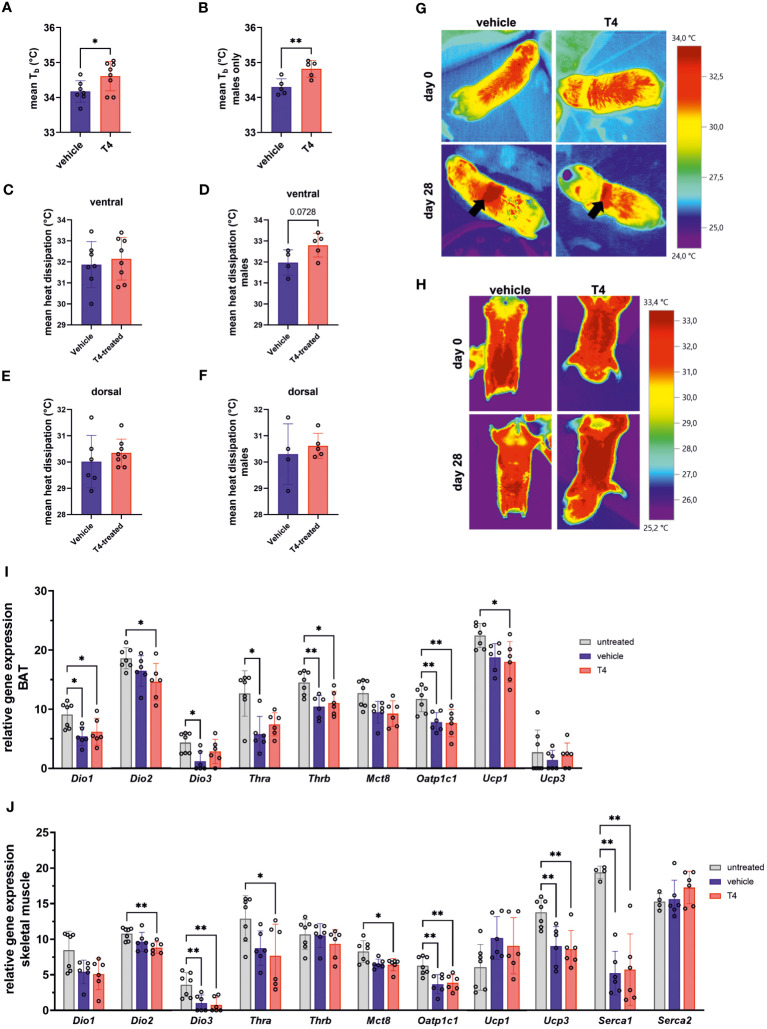
Treatment effects on thermoregulation. Mean T_b_ of **(A)** female and male Ansell’s mole-rats and **(B)** male Ansell’s mole-rats on the last day of treatment, measured with implanted temperature loggers (Unpaired *t*-test, N=7–8). Mean ventral heat dissipation at the end of treatment in **(C)** female and male Ansell’s mole-rats and **(D)** male Ansell’s mole-rats only (Unpaired t-test, N=7–8 all animals and N=4–5 males only). Mean dorsal heat dissipation at the end of treatment in **(E)** female and male Ansell’s mole-rats and **(F)** male Ansell’s mole-rats only (Unpaired *t*-test, N=6–8 all animals and N=4–5 males only). Representative images of **(G)** dorsal and **(H)** ventral heat dissipation in vehicle- and T4-treated animals before and after treatment for four weeks. Arrows indicate areas shaved for osmotic pump implantation, which were excluded from analysis. Relative gene expression levels per 100 pg cDNA of **(I)** BAT and **(J)** skeletal muscle, representing two major thermoregulatory organs contributing to facultative thermogenesis, of untreated, vehicle- and T4-treated animals (one-way ANOVA followed by Tukey’s multiple comparisons test or Kruskal-Wallis test followed by Dunn’s multiple comparisons test, N=6–7, except for *Serca1* and *Serca2*: N=4–6). Gene expression of untreated Ansell’s mole-rats was previously published (28) and is publicly available (37). Data are presented as means ± SD. *p ≤ 0.05 and **p< 0.01.

THs increase obligatory thermogenesis via upregulation of metabolic rate and BAT activity. Thus, we hypothesized that T4 treatment would cause both, increased T_b_ and increased heat dissipation in mole-rats. First, we analyzed T_b_ monitored by the implanted temperature loggers and determined the mean T_b_ in a time frame of 24 h prior to terminal sampling. Prior to statistical analysis, we observed that treatment effects in female mole-rats were not as pronounced as in males. Therefore, we first calculated a multiple linear regression model with treatment and sex as explanatory variables and estimated the best fitting model. According to the best fitting, simpler model (model 2; **Table 2**), both, T4 treatment and sex exerted significant effects on T_b_. Thus, we calculated one t-test including all animals (**Figure 4A**) and another t-test including only male mole-rat data (**Figure 4B**). Both scenarios revealed significantly higher T_b_ in T4-treated animals, but the treatment effect was stronger in males. Next, we analyzed heat dissipation using an infrared camera. We compared the mean body surface temperature on the ventral and dorsal sides of the animals on the last day of treatment (**Table 3**; **Figures 4C-H**). Again, to account for sex differences, we calculated a multiple linear regression model with treatment and sex as explanatory variables. In none of the models, we found a treatment effect, but sex exerted a significant effect in ventral heat dissipation in the best fitting model (model 2; **Table 3**). In line, ventral and dorsal heat dissipation did not significantly differ between vehicle- and T4-treated animals. After excluding data from female mole-rats, T4-treated animals showed a trend towards higher ventral heat dissipation (*p*=0.073). In BAT, gene expression of *Dio2* and *Ucp1*, which are both essential for BAT thermogenesis, were significantly downregulated in T4-treated animals, while *Dio1* and *Oatp1c1* were significantly downregulated in vehicle- and T4-treated animals compared to untreated controls (**Figure 4I**). In skeletal muscle, *Dio2* expression was significantly downregulated in T4-treated animals, but most other tested genes, including *Ucp3* and *sarcoplasmic/endoplasmic reticulum calcium ATPase* 1 (*Serca1*), which are involved in thermogenesis, were downregulated in both, vehicle- and T4-treated animals compared with untreated control animals (**Figure 4J**).

**Table 2:** Summary of multiple linear regression calculated to determine the best predictor of differences in mean core body temperature (AIC, Akaike information criterion).

model	factors	*p*	AIC	Probability of correct model (%)
1	treatment	0.017	-24.92	9.45
	sex	0.096		
	treatment × sex	0.749		
2	treatment	0.006	-29.44	90.55
	sex	0.007		

**Table 3:** Summary of multiple linear regression models calculated to determine the best predictor of differences in mean heat dissipation (AIC, Akaike information criterion).

Mean heat dissipation (ventral)				
model	factors	*p*	AIC	Probability of correct model (%)
1	treatment	0.087	0.63	11.66
	sex	0.078		
	treatment × sex	0.4083		
2	treatment	0.109	-3.42	88.34
	sex	0.002		
Mean heat dissipation (dorsal)				
model	factors	*p*	AIC	Probability of correct model (%)
1	treatment	0.514	3.010	7.52
	sex	0.194		
	treatment × sex	0.874		
2	treatment	0.337	-2.009	92.48
	sex	0.064		

### Treatment effects on the hypothalamic-pituitary-adrenal axis

**Figure 5:**
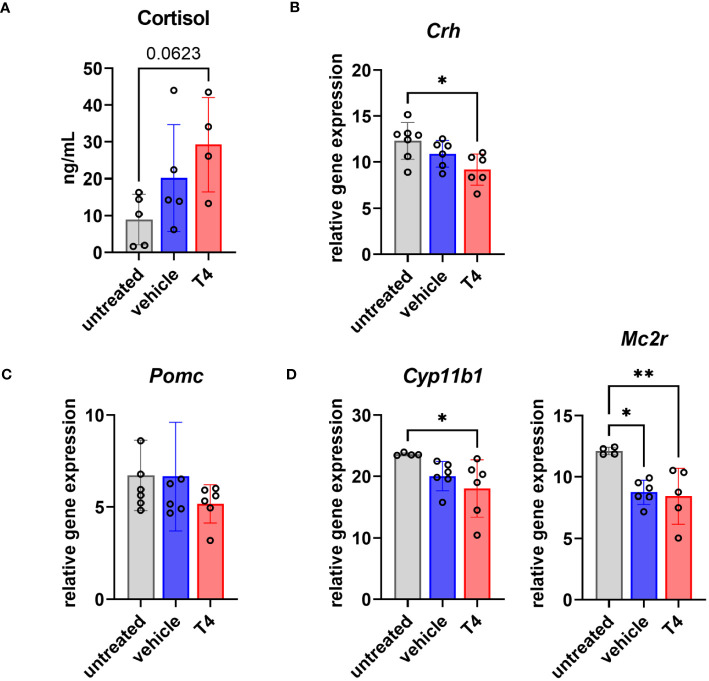
Treatment effects on the hypothalamus-pituitary-adrenal (HPA) axis. **(A)** Serum cortisol concentrations of untreated Ansell’s mole-rats as well as vehicle-treated and T4-treated Ansell’s mole-rats 4 weeks after pump implantation (one-way ANOVA followed by Tukey’s multiple comparisons test, N=4–5). Relative gene expression levels per 100 pg cDNA of **(B)** *Crh* in the hypothalamus, **(C)** *Pomc* in the pituitary, and **(D)** *Cyp11b1* and *Mc2r* in the adrenal gland from untreated, vehicle- and T4-treated animals (one-way ANOVA followed by Tukey’s multiple comparisons test, N=6–7). Gene expression of untreated Ansell’s mole-rats was previously published (28) and is publicly available (37). *p ≤ 0.05 and **p < 0.01.

In addition to the HPT axis, the hypothalamic-pituitary-adrenal (HPA) axis is also involved in positively regulating energy expenditure. Both endocrine systems are thus closely intertwined. Therefore, we also assessed T4 treatment effects on the HPA axis. We found 2-fold and 3-fold higher mean serum cortisol concentrations in vehicle- and T4-treated animals, respectively, after 4 weeks of treatment (**Figure 5A**). Furthermore, we measured gene expression of four central components of the HPA axis in the hypothalamus (corticotropin-releasing hormone coded by *Crh*; **Figure 5B**), the pituitary (proopiomelanocortin coded by *Pomc*; **Figure 5C**), and the adrenal gland (steroid 11β-hydroxylase coded by *Cyp11b1* and ACTH/melanocortin 2 receptor coded by *Mc2r*; **Figure 5D**). While *Crh* and *Cyp11b1* expression was significantly downregulated only in T4-treated animals, *Mc2r* expression was significantly downregulated in vehicle- and T4-treated animals compared to untreated controls.

## Discussion

Living in subterranean burrow systems comes with ecophysiological challenges, including the risk of overheating, temporary hypoxia and hypercapnia, and scarce food availability. Low RMR and low T_b_ are considered important convergent ecophysiological adaptations to these ecological challenges in African mole-rats (3, 6), but the upstream regulation of these important physiological traits was unknown thus far. We have previously described peculiarities of the TH system of naked and Ansell’s mole-rats involving serum TH concentrations, the HPT axis, and peripheral TH metabolism by deiodinases (27, 28, 42). Because THs are important regulators of metabolic rate and thermoregulation (11), we hypothesized that the bathyergid TH system serves as the upstream regulator of low RMR as well as low T_b_. As expected, T4 treatment of Ansell’s mole-rats yielded upregulation of T4, T3, and rT3. On the HPT axis, we also found pronounced treatment effects in T4-treated animals compared with vehicle-treated animals, including differential gene expression of TH regulators in the hypothalamus, significantly lower *Tshb* gene expression in the pituitary, and significantly lower follicular cell height and *Tpo* expression in the thyroid, all indicative of downregulated endogenous TH synthesis. On the other hand, other expected hallmarks of HPT axis response to elevated TH levels such as downregulation of *Trh* expression in the hypothalamus were lacking. It has often been questioned whether the negative feedback loop of the HPT axis is functional in mole-rats, because of the extraordinarily low serum T4 concentrations in naked and Ansell’s mole-rats (26–28). Our data strongly indicate that TH synthesis is negatively regulated by a pituitary-thyroid axis, but it is still questionable to which extent the hypothalamus contributes to negative feedback regulation. Although T4 treatment did not influence *Trh* expression, we found significant downregulation of *Dio2*, *Mct8*, and *Oatp1c1*, suggesting that the hypothalamus reacts to circulating T4 in Ansell’s mole-rats. Furthermore, T4-treated animals exhibited significantly decreased TT4 to TT3 conversion rates and significantly higher serum rT3 concentrations, the inactive metabolite of T4, representing important peripheral mechanisms of TH regulation. Unexpectedly, animals implanted with vehicle-filled pumps also showed a slight upregulation of serum TT4, which was significant but still about 5-fold lower compared to T4-treated animals. On the other hand, serum concentrations of fT4 and fT3, which represent the biologically-active fractions, were not significantly altered in vehicle-treated animals. We strongly assume that this slight upregulation of endogenous TH synthesis was caused by the surgical procedures required to implant the osmotic pump and the temperature logger. THs have been reported to support wound healing (43–45), and, albeit not fully understood, THs and the immune system exert a bidirectional crosstalk (46, 47). Consequently, wound healing and the upregulated immune response post-surgery may have caused an upregulation of endogenous T4 synthesis observed in vehicle-treated animals. Another potential explanation for elevated TT4 in vehicle-treated animals is directly linked to these aspects. The surgeries that were conducted in both experimental groups might have activated the HPA axis, as a stress-response. Accordingly, we found 2- and 3-fold higher mean serum cortisol concentrations at the end of the 4-week treatment period in vehicle- and T4-treated animals, respectively, which represents the major glucocorticoid in African mole-rats (48). As we measured cortisol only after 4 weeks post-surgery, we were not able to analyze a hormonal response directly after surgery. On gene expression level, we found that *Crh* and *Cyp11b1* were downregulated in T4-treated animals and *Mc2r* was downregulated in both treatment groups compared to untreated control animals. This downregulation of central components of the HPA axis on genetic level likely represents a negative feedback regulation counteracting elevated cortisol levels (49, 50). THs and glucocorticoids are hormones that strongly affect energy expenditure, and as such a bidirectional crosstalk was described between the HPT and the HPA axis that positively regulate TH and glucocorticoid concentrations (51, 52). Therefore, elevated cortisol concentrations triggered by the surgery and the wound healing process may have activated the HPT axis, resulting in elevated TT4 concentrations also in vehicle-treated animals.

Many of the genes tested were not differentially expressed in most tissues between vehicle- and T4-treated animals, but when compared with untreated animals, we found profound differences. On the other hand, treatment effects were much more pronounced between vehicle- and T4-treated animals at the physiological level, e.g. in body weight and thermoregulation. We can conclude from these data that TH-related gene expression is very sensitive to small changes in circulating TH, because slight upregulation of TT4 in vehicle-treated animals resulted in similar effects on gene expression level as observed in T4-treated animals with much higher circulating TH concentrations. Alternatively, these findings suggest that a higher T4 dose might be necessary to surpass the ability of the mole-rats’ TH system to buffer these increases. Still, expression of *Tshb* in the pituitary, which codes for the β-subunit of TSH, was highly downregulated exclusively in T4-treated animals, providing further evidence that negative feedback regulation on the pituitary-thyroid level is functional in mole-rats and that endogenous TH synthesis was largely downregulated in T4-treated but not vehicle-treated animals. These conclusions are further supported by lower thyroid follicle cell height and the trends towards lower DIO1 and DEHAL1 activity in T4-treated animals, which are indicative of lower thyroid activity.

In the periphery, we were particularly interested in TH-related effects on RMR and thermogenesis. Contrary to expectations, both, vehicle- and T4-treatment resulted in a significant upregulation of msRMR. These findings suggest that even a slight upregulation of serum T4 as observed in vehicle-treated animals is sufficient to significantly increase msRMR. To our surprise, T4-treatment did not yield msRMR beyond the levels observed in vehicle-treated animals, for which there are two potential explanations. First, the RMR of Ansell’s mole-rats exhibits only a narrow range, with a maximum RMR not exceeding the maximum observed in the present study. Second, TH action could be downregulated at tissue level in metabolically active organs such as liver and kidney. Third, elevated cortisol concentrations in both treatment groups could have masked TH effects on msRMR. Metabolic rate outside the thermoneutral zone or during digging can increase by about 5-fold compared to RMR (7, 53), suggesting that RMR in T4-treated animals has the potential to increase beyond the levels observed in the present study, especially when compared to vehicle-treated animals, because T4 concentrations were several times higher in T4-treated animals. This makes the first explanatory approach unlikely. The second explanatory approach is supported by higher serum rT3 concentrations and a lower TT3/TT4 ratio, indicating higher T4 inactivation and lower conversion of T4 into the active T3, respectively. In a previous study, we measured hepatic DIO1 activity in untreated mole-rats, which was very low and even not detectable in 4 out of 7 animals (28). Although hepatic DIO1 activity did not differ between vehicle- and T4-treated animals in the present study, it was obviously increased compared to untreated animals, providing a possible mechanism for the limited upregulation of RMR. DIO1 is responsible for 5’-deiodination of rT3 and T4, with a higher preference for rT3 as a substrate (54). Thus, hepatic and renal DIO1 activity could be responsible for either T4 inactivation, rT3 cleavage, or both. In mice, hyperthyroidism leads to increased expression of *Dio1* in the kidney as well as *Dio1* and *Spot14* in the liver, both TH-responsive genes (55, 56). In Ansell’s mole-rats, these genes were not differentially regulated in either the kidney or liver in T4-treated animals, suggesting that TH signaling is effectively downregulated despite higher serum TH concentrations. Of note, the treatment dose used in the present study yielded an average increase in TT4, fT4, and fT3 of about 25-fold, 9-fold, and 2-fold, respectively, which is many times higher than in hyperthyroid mice treated with T4 (56, 57). Thus, our data point towards an extraordinary capability of mole-rats to effectively downregulate TH signaling at tissue level despite very high serum TH concentrations. Moreover, higher rates of TH inactivation as well as a lack of TH activation by DIO2 in metabolically demanding organs such as liver and kidney limit the capacity of THs to upregulate RMR in T4-treated animals. This suggests that a low RMR is a selective driver for the development of mechanisms to actively downregulate TH action, resulting in a wide range in which mole-rats can tolerate changes in serum TH to stabilize RMR at a low level. In future studies, higher T4 concentrations or T3 treatment might be required to yield also stable changes in physiological parameters such as RMR. The second explanatory approach is well complemented by the third explanatory approach. Cortisol was previously reported to increase RMR and energy expenditure in mammals (58, 59). Thus, increased cortisol in vehicle- and T4-treated animals could have masked increased msRMR in T4-treated animals. An involvement of cortisol is also supported by depleted glycogen stores in liver and skeletal muscle in both treatment groups compared with untreated animals, which represents a typical stress response. On the other hand, we also found that T4-treated animals lost body weight during treatment, which was not observed in vehicle-treated animals despite similar msRMR and depleted glycogen stores. A bi-directional crosstalk between the HPA and HPT axis might explain this difference in body weight, as these effects could have mutually amplified each other in T4-treated animals. However, it cannot be excluded that T4 treatment reduced calorie intake or the digestibility coefficient of the animals, which could serve as an alternative explanation for significant weight loss by T4 treatment compared to vehicle treatment despite similar msRMR. In light of the *cost of burrowing hypothesis*, energy stores are critical to compensate for the high energetic costs of burrowing (9). This, in turn, emphasizes the need to keep TH and presumably cortisol concentrations low to maintain energy stores. To date, only a few studies have investigated THs in rodent species that inhabit underground burrows other than bathyergid rodents. From these studies, we can conclude that at least Merriam’s kangaroo rats [*Dipodomys merriami* (60)], blind mole-rats [*Spalax* sp. (61)], and Mongolian gerbils [*Meriones unguiculatus* (62, 63)] have low RMR associated with peculiarities in circulating TH concentrations. Thus, our data, in conjunction with previous studies, points towards convergent ecophysiological adaptations to the subterranean habitat involving THs.

Although we did not find differences in msRMR between vehicle- and T4-treated animals, T_b_ was significantly upregulated in T4-treated animals compared to vehicle-treated animals in a similar extent reported for thyrotoxicosis mice (56). According to the *thermal stress hypothesis*, RMR is kept low in subterranean mammals because metabolism produces heat, which is known as obligatory thermogenesis. Since msRMR did not differ between vehicle- and T4-treated animals, it is likely that processes other than obligatory thermogenesis were responsible for the upregulation of T_b_ in Ansell’s mole-rats. Besides obligatory thermogenesis, THs are involved in the regulation of facultative thermogenesis, namely shivering and non-shivering thermogenesis, through central and peripheral mechanisms involving UCP1 and possibly UCP3 (16, 64, 65). The present gene expression data allow us interesting insights: unexpectedly, we found that the T3 target gene *Ucp1* was significantly downregulated in T4-treated animals and the T3-target genes *Ucp3*, and *Serca1* were downregulated in vehicle- and T4-treated compared with untreated animals in skeletal muscle, suggesting that TH signaling was downregulated in these tissues. Expression of *Dio2*, coding for the activating deiodinase, was downregulated in BAT and skeletal muscle of T4-treated animals but not in vehicle-treated animals. Since DIO2 activity is required to provide sufficient T3 by T4 deiodination in BAT (66), downregulation of *Dio2* expression in T4-treated animals was likely responsible for the reduced *Ucp1* and *Ucp3* expression. Therefore, we assume that there must have been another source of the increased thermogenesis in T4-treated animals that could not be identified in the present study. In line with our findings, TH-induced increase of T_b_ was already reported to be independent of UCP1 in mice, suggesting that TH-regulated thermogenesis is BAT-independent (56, 67). Instead, THs are likely to upregulate the hypothalamic body temperature setpoint with implications for cardiovascular function (67, 68).

Interestingly, we found that treatment effects on T_b_ and heat dissipation were sex-dependent, and female mole-rats showed less change in response to treatments. Estrogens have previously been shown to modulate several aspects of thermoregulation via central and peripheral mechanisms (69, 70), leading to downregulation of T_b_ (71, 72). Furthermore, an interaction between estrogens and thyroid hormones with implications for e.g. seasonal reproduction has been reported (73). These direct effects on thermoregulation and a potential modulation of TH signaling by estrogens could be a possible explanation for the less pronounced effect of treatment in female mole-rats with respect to thermoregulation. In African mole-rats, the venter is considered the main thermal window for enhancing heat dissipation by conductance (74, 75). However, we did not observe a significant increase of ventral heat dissipation in T4-treated animals despite higher T_b_ except for a trend in male mole-rats. Ansell’s mole-rats also exhibit behavioral mechanisms to reduce thermoregulatory costs, such as huddling or by choosing appropriate ambient or surface temperatures (76, 77). Therefore, avoidance of huddling or resting at lower surface temperatures, could have been alternative strategies to increase heat dissipation in T4-treated animals. This could explain why mean T_b_ observed in T4-treated Ansell’s mole-rats (34.6°C) was still within the physiological range of this species, which is between 33.8 and 36.1°C (3), suggesting that Ansell’s mole-rats were still able to maintain T_b_ below a harmful threshold despite strong upregulation of TT4. On the other hand, we cannot infer from the present data to which extent T_b_ could be further upregulated in subterranean burrows where heat dissipation is less efficient. In their natural habitat, Ansell’s mole-rats might already face increased risk for overheating under the same conditions. Taken together, in light of the *thermal stress hypothesis*, we can conclude that low T_b_ in this mole-rat species is not simply a consequence of low RMR but is directly linked to serum T4.

## Conclusion

Low RMR and low T_b_ are convergent physiological features shared by many subterranean rodents. However, the upstream regulation of this trait was previously unknown. Since the first description of the extraordinarily low T4 concentrations in naked mole-rats and Ansell’s mole-rats (26, 27), the TH system of mole-rats has been a promising candidate to elucidate these upstream mechanisms, but experimental evidence has been lacking. In the present study, we could link RMR and T_b_ with serum T4 concentrations and provided evidence for compensatory mechanisms that might reduce TH action at tissue level to limit harmful increases of RMR and T_b_. We can infer from these data that the TH system of Ansell’s mole-rats most likely serves as the upstream regulator of ecophysiological adaptations to the subterranean habitat.

## Data availability statement

The original contributions presented in the study are included in the article/**Supplementary Material**, further inquiries can be directed to the corresponding author/s.

## Ethics statement

The animal study was approved by North Rhine-Westphalia State Environmental Agency (LANUV). The study was conducted in accordance with the local legislation and institutional requirements.

## Author contributions

PG: Writing – review & editing, Writing – original draft, Visualization, Methodology, Investigation, Formal Analysis, Data curation. SB: Writing – review & editing, Methodology, Investigation. CF: Writing – review & editing, Methodology, Investigation, Formal Analysis. KR: Writing – review & editing, Resources, Methodology, Investigation, Formal Analysis. AH: Writing – review & editing, Investigation, Formal Analysis. JK: Writing – review & editing, Validation, Resources, Methodology, Formal Analysis. YH: Writing – review & editing, Writing – original draft, Visualization, Validation, Supervision, Resources, Project administration, Methodology, Investigation, Funding acquisition, Formal Analysis, Data curation, Conceptualization.
