# Supplementary material for: Low thyroxine serves as an upstream regulator of ecophysiological adaptations in Ansell’s mole-rats
Source: Front Endocrinol (Lausanne). 2024 Mar 19;15:1329083. doi: 10.3389/fendo.2024.1329083 (PMC10985354; doi:10.3389/fendo.2024.1329083)
Supplement: Supplementary file 1 [file Table_1.docx]

**Table S1: Information on animals used in the present study.** Abbreviations: VH – vehicle, T4 – thyroxine, UT – untreated, F – female, M – male.

|  | Treatment | Body weight (g) | Sex | Age (months) |
| --- | --- | --- | --- | --- |
| 1 | VH | 113.2 | M | 31 |
| 2 | VH | 60.2 | F | 25 |
| 3 | VH | 100.6 | M | 26 |
| 4 | VH | 103.3 | F | 21 |
| 5 | VH | 82 | M | 22 |
| 6 | VH | 119 | M | 33 |
| 7 | VH | 91 | M | 21 |
| 8 | T4 | 99.5 | F | 28 |
| 9 | T4 | 88.2 | F | 21 |
| 10 | T4 | 136 | M | 33 |
| 11 | T4 | 130.2 | M | 18 |
| 12 | T4 | 97.6 | M | 18 |
| 13 | T4 | 100.0 | M | 18 |
| 14 | T4 | 94.3 | F | 38 |
| 15 | T4 | 121 | M | 26 |
| 16 | UT* | 54.3 | F | 30 |
| 17 | UT* | 93.5 | F | 14 |
| 18 | UT* | 62.3 | M | 12 |
| 19 | UT* | 78 | M | 12 |
| 20 | UT* | 59.6 | F | 19 |
| 21 | UT* | 70.3 | M | 19 |
| 22 | UT* | 53.9 | M | 11 |

* Data of untreated Ansell’s mole-rats were included in gene expression analyses. These data were compiled as part of a previous study (1) and are publicly available (2).

**References**

1. Gerhardt P, Begall S, Frädrich C, Renko K, Hildebrandt TB, Holtze S, et al. Comparative analysis of thyroid hormone systems in rodents with subterranean lifestyle. Sci Rep. 2023;13(1):3122.

2. Gerhardt P, Begall S, Frädrich C, Renko K, Hildebrandt TB, Holtze S, et al. Gerhardt et al_Gene expression data.xlsx. Figshare. 2022.
